# TREM2 ectodomain and its soluble form in Alzheimer’s disease

**DOI:** 10.1186/s12974-020-01878-2

**Published:** 2020-07-07

**Authors:** Jiaolong Yang, Zhihui Fu, Xingyu Zhang, Min Xiong, Lanxia Meng, Zhentao Zhang

**Affiliations:** Department of Neurology, Renmin Hospital of Wuhan University, Wuhan, 430060 China

**Keywords:** Triggering receptor expressed on myeloid cells 2, Microglia, Alzheimer’s disease, Neuroinflammation

## Abstract

Triggering receptor expressed on myeloid cells 2 (TREM2) is a receptor mainly expressed on the surface of microglia. It mediates multiple pathophysiological processes in various diseases. Recently, TREM2 has been found to play a role in the development of Alzheimer’s disease (AD). TREM2 is a transmembrane protein that is specifically expressed on microglia in the brain. It contains a long ectodomain that directly interacts with the extracellular environment to regulate microglial function. The ectodomain of TREM2 is processed by a disintegrin and metalloprotease, resulting in the release of a soluble form of TREM2 (sTREM2). Recent studies have demonstrated that sTREM2 is a bioactive molecule capable of binding ligands, activating microglia, and regulating immune responses during the AD continuum. Clinical studies revealed that sTREM2 level is elevated in cerebrospinal fluid (CSF) of AD patients, and the sTREM2 level is positively correlated with the levels of classical CSF biomarkers, namely t-tau and p-tau, indicating that it is a reliable predictor of the early stages of AD. Herein, we summarize the key results on the generation, structure, and function of sTREM2 to provide new insights into TREM2-related mechanisms underlying AD pathogenesis and to promote the development of TREM2-based therapeutic strategy.

## Background

Alzheimer’s disease (AD), as the most common neurodegenerative disease, has been plaguing aging individuals since 1906 when it was first described by the German psychiatrist Alois Alzheimer [1]. It has been reported that dementia and the other disabilities caused by AD are the sixth leading cause of death in the USA [2]. Extracellular deposits of amyloid β (Aβ), which produce amyloid plaques, and intracellular aggregates of tau, which generate neurofibrillary tangles, are the two major hallmarks of the disease [3]. Much effort has been devoted to identifying the underlying mechanisms of the disease. It is believed that AD is a multifactorial pathology induced by a combination of age, genetic factors, and environmental factors [4–6]. Recently, genome-wide association studies identified that triggering receptor expressed on myeloid cells 2 (TREM2) is one of the strongest genetic risk factors for AD, following *APP*, *PSEN1*, and *APOE* [7, 8]. Recent studies have also indicated that APOE gene participates in the development of AD in a TREM2-dependent manner [9]. Thus, we saw a burst of studies on the role of TREM2 in AD onset and development.

As a member of the immunoglobulin superfamily, TREM2 is a type I transmembrane protein that is exclusively expressed by microglia in the brain. It plays essential roles in cell survival, cell proliferation, and phagocytosis. By regulating the function of microglia, it maintains the homeostasis of the central nervous system (CNS) [10]. TREM2 is a transmembrane protein and functions as a receptor on the cell membrane. It binds ligands through its ectodomain to activate intercellular signaling pathways, which control innate immune responses. Various ligands including exogenous pathogens and endogenous proteins can interact with the ligand-binding domain of TREM2, thereby activating microglia and promoting phagocytosis through the TREM2–DAP12-dependent pathway [11]. Furthermore, in vitro and in vivo studies have demonstrated that its extracellular domain can be cleaved by different sheddases to generate a soluble form of TREM2 (sTREM2), which functions independently of TREM2 to regulate interactions between neurons and the surrounding microenvironment [12]. Another source of sTREM2 is the non-proteolytic-mediated secretion of some TREM2 isoforms, which may have arisen from alternative splicing of the transcript, into the extracellular space [13]. It has been reported that sTREM2 mediates the biological functions of TREM2 and regulates multiple pathophysiological processes. Of note, the level of sTREM2 in the cerebral spinal fluid (CSF) has been reported to be a reliable predictor of AD [14]. As sTREM2 is primarily generated by the proteolytic cleavage of the ectodomain and is considered to be identical to the ectodomain of full-length TREM2 in terms of amino acid sequence, we will focus on the ectodomain of TREM2 and the product of its proteolytic cleavage, namely sTREM2, in the molecular mechanisms of AD as well as in the discovery of new therapeutic targets.

## TREM2 gene-structure

### Structure of hTREM2

**Fig. 1:**
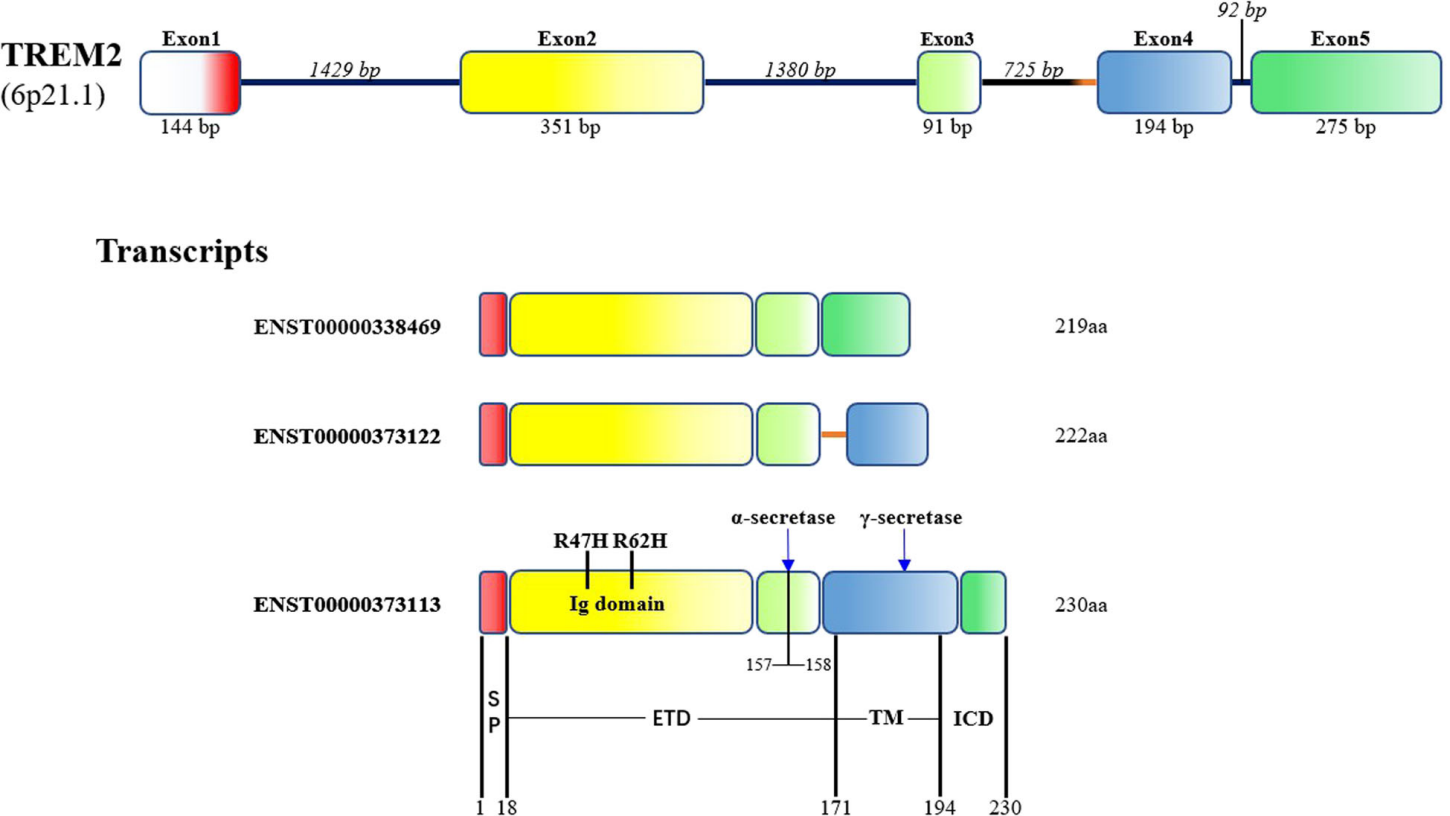
Schematic representation of the human TREM2 gene and protein. TREM2 gene showing exonic (numbered boxes) and intronic (line) regions and sizes. The transcripts are shown with translated regions, and different colors represent different coding sequences of the transcription template. The three isoforms (ENST00000373113, ENST00000373122, and ENST00000338469) have the same exons 1, 2, and 3 coding regions but different exons 4 and 5 coding regions. ENST00000373122 contains a portion of the intronic sequence. The bottom panel shows the TREM2 protein, including the signal peptide (SP), immunoglobulin (Ig) domain, extracellular region (ETC), and transmembrane region (TM). Arrows represent α-secretase and γ-secretase cleavage sites, respectively. α-Secretase (ADAMs) cleaves TREM2 at His157−Ser158 site. R47H and R62H are two common AD-associated variants occurring in the Ig domain. aa, amino acid

TREM2 is encoded by the *TREM2* gene located on human chromosome 6*p*21.1 [15]. The TREM2 protein is comprised of an extracellular ectodomain (1−172 amino acids [aa]), a transmembrane domain (173−195 aa), and an intracellular domain (196−230 aa) [16]. Cell signaling is collectively mediated by the three regions of the TREM2 protein, with the ectodomain binding extracellular ligands and the intracellular domain binding intermediate signaling proteins to ensure completion of a specific cellular event. In 2014, Jin and colleagues first identified three alternatively spliced TREM2 transcripts (ENST00000373113, ENST00000373122, and ENST00000338469) in the human brain by polymerase chain reaction (PCR) using transcript-specific primers [17]. Interestingly, the three TREM2 isoforms are identical to full-length TREM2 in terms of the signal peptide sequence (1−18 aa), the immunoglobulin-like extracellular domain sequence (19−134 aa), and the short stalk sequence (135−161 aa), indicating that the homologous sequences, which are located within the canonical TREM2 ectodomain (Fig. 1), probably take part in critical functions mediated by TREM2 signaling.

Furthermore, the ectodomain of TREM2 is susceptible to various post-translational modifications, such as glycosylation and sulfidation [18, 19], indicating that the ectodomain is critical for protein function. For example, glycosylation of the V-type Ig domain of TREM2 may promote intramolecular interactions, as the formation of two disulfide bonds between residues C36 and C110 and another bond between residues C51 and C60 can create a specific ligand-binding site [19]. Therefore, modifications of the TREM2 ectodomain can affect its binding function and regulate its conformation on cell membrane.

### TREM2 isoforms

Several important differences have been reported for the three alternatively spliced transcripts of TREM2 [13, 17]. Canonical transcript ENST00000373113, as the longest TREM2 transcript, is most highly expressed in the brain. It contains five exons, and the protein consists of 230 amino acids [20]. This TREM2 isoform has a transmembrane domain, which can be cleaved by sheddases, such as a disintegrin and metalloproteases (ADAMs) [21, 22]. Two additional TREM2 isoforms are supposed to encode soluble proteins due to the lack of transmembrane domains (encoded by exon 4), and both TREM2 isoforms are secreted via a non-proteolytic mechanism. It is worth noting that transcript ENST00000338469, as the shortest TREM2 transcript, has been widely studied because it is a soluble protein that has been detected in the CSF and interstitial space [13, 23]. This TREM2 transcript is also regarded as a reliable predictor of AD before the onset of clinical symptoms [24]. However, the function of transcript ENST00000373122 is still largely unknown.

## Origins of sTREM2

sTREM2 is generated either by the proteolytic cleavage of canonical TREM2 or the secretion of alternatively spliced transcripts, with the most common form involving shedding of the ectodomain by specific proteases [12]. Sheddases of the metalloprotease family (e.g., ADAM10 and ADAM17) have been reported to cleave TREM2 at the stalk, thereby liberating the Ig domain and generating sTREM2 [25]. The results of a mass spectrometry study indicated that both ADAM10 and ADAM17 cleave TREM2 at His157−Ser158 in human cultured macrophages [26]. Interestingly, it has been reported that the H157Y TREM2 gene mutation promotes its shedding by ADAM and is associated with an increased risk of AD [27], revealing the clinical relevance of this cleavage site. Both ADAM10 and ADAM17 appear to be equally important in the cleavage of TREM2 [21, 28]. Following ADAMs cleavage, the resultant transmembrane fragment undergoes additional proteolytic cleavage by γ-secretase [12]. It is still unknown whether TREM2 can be cleaved by other proteases.

Alternative splicing of the *TREM2* transcript is responsible for the secretion of two sTREM2 isoforms (ENST00000338469 and ENST00000373122). Both isoforms have been detected in the CNS [13, 17]. The shortest isoform of TREM2 (ENST00000338469) completely lacks exon 4, whereas the other isoform contains exon 4 but lacks the proper transmembrane domain (Fig. 1). By investigating RNA-Seq data of brain tissues from AD patients, one previous study demonstrated that the expression level of transcript (ENST00000338469) was significantly lower than that of the canonical transcript (ENST00000373113), and only account for about 25% of sTREM2 in the brain [13]. Presently, it is still unclear whether ENST00000373122 is embedded within the cell membrane [17]. Further studies are needed to understand the roles of these TREM2 isoforms in AD.

## Localization of sTREM2

In general, sTREM2 is released into the extracellular or luminal space [25]. Depending on the cellular context, sTREM2 secretion varies, and it can settle into different extracellular spaces. For instance, microglial TREM2 is cleaved and secreted into the brain parenchyma [29, 30], whereas sTREM2 is derived from monocytes and secreted into the blood [21]. The presence of sTREM2 in the extracellular space and CSF during the onset of AD suggests that sTREM2 participates in a broad range of functions [31]. For example, sTREM2 can settle around necrotic neurons, abnormal protein aggregates, or foreign microorganisms [28, 32], where it may bind diverse receptors to activate innate immune responses and trigger disease conditions. On the other hand, the uncontrolled proteolytic cleavage of TREM2 results in a very high level of sTREM2, which can disturb the blood−brain barrier and result in its diffusion into the CSF [33, 34]. Furthermore, small quantities of sTREM2 in the plasma may also diffuse through damaged blood vessels and enter into the brain parenchyma [35], which may partly explain why the sTREM2 level is elevated in cases of AD as well as other neurodegenerative diseases, such as multiple sclerosis (MS), frontal temporal dementia (FTD), and Lewy body dementia (LBD) [24, 36, 37]. Taken collectively, these data indicate that sTREM2 is generated via proteolysis in local tissues and then can enter the CSF after passing through the damaged blood–brain barrier.

The sTREM2 level in the CSF, which is elevated during the AD continuum, follows a dynamic course of changes [23, 38], and these changes are instrumental in diagnosing and staging AD. Interestingly, several studies have reported an elevated sTREM2 level in some patients as early as 5 years before the onset of AD [39, 40]. Furthermore, the elevated sTREM2 level in the CSF of AD patients is correlated with the levels of other well-established neurodegenerative markers, such as t-tau/p-tau, and Aβ42 [41]. However, there are still disputes about whether the diagnostic significance of sTREM2 in AD is influenced by TREM2 mutants. For example, R47H mutation carriers showed increased sTREM2 expression, whereas R62H mutation carriers showed no significant change on sTREM2 level in the CSF compared to non-carriers [42]. Larger sample size is needed to verify whether sTREM2 is increased in AD patients who are not TREM2 mutant carriers.

The presence of sTREM2 in the CSF of AD patients is believed to be the result of microglial activation [39, 43], which is closely associated with neuroinflammation during the early stages of the disease [44]. This idea is supported by the fact that the sTREM2 concentration increases after amyloid deposition and subsequent neuronal injury [40]. Furthermore, the expression level of sTREM2 in CSF was reported to be positively correlated with that of CSF monocyte chemoattractant protein 1, a putative microglial marker, at the early stage of AD progression [45]. However, the sTREM2 level in the CSF is not always elevated in AD. A recent meta-analysis revealed that the sTREM2 level was significantly higher at an early stage of AD than that at a late stage [46]. Therefore, further studies are needed to collect additional data (e.g., disease type, stage, and pathological features) to understand the association between sTREM2 and AD progression.

Presently, the clinical relevance of an elevated serum sTREM2 level in AD is unknown [34]. Several studies have reported the sTREM2 level in the peripheral blood to be higher in AD patients than that in healthy individuals [47, 48], whereas other investigators reported no significant difference [24, 46]. This discrepancy may be attributed to the designation of groups, as important patient characteristics (i.e., gender, age, and disease stage) may affect the results. Furthermore, the origins of serum sTREM2 are still under study [49]. For example, monocyte-derived dendritic cells (moDCs) express a high level of membrane-bound TREM2, which is susceptible to proteolysis by sheddases [22]. Other cells, such as osteoclasts, can also produce and release sTREM2 into the bloodstream, making it difficult to assess the influence of serum sTREM2 in AD development. Finally, TREM2 variant carriers further complicate our understanding of this molecule, as some TREM2 mutants have a strong impact on the sTREM2 level in myeloid cells [42]. TREM2 variants can upregulate or downregulate protein cleavage or influence the shedding of sTREM2 into body fluids [18]. Further studies are needed to investigate the metabolism of sTREM2 in the peripheral blood.

In addition, the extracellular distribution of other sTREM2 isoforms, ENST00000373122 and ENST00000338469, should not be ignored. ENST00000338469 was found to exist in the extracellular space and CSF [50]. One study of 345 specimens reported that up to 25% of the extracellular sTREM2 was derived from the expression of ENST00000338469 instead of the cleavage of canonical TREM2 [13]. By contrast, few expression studies have been performed on ENST00000373122. It should be noted that most of the previous studies on the concentration of sTREM2 in the CSF did not differentiate the isoforms of sTREM2 [23, 24, 42]. For example, Rauchmann et al. conducted two ELISA assays at two different laboratories to measure the concentration of CSF sTREM2. However, the capture and detection antibodies used in both laboratories were against the conserved extracellular amino acids of different isoforms of sTREM2 [23]. In this case, these approaches could not distinct sTREM2 variants in CSF. Thus, more specific antibodies against distinct sTREM2 isoforms based on their unique epitopes should be developed to identify the proportion of different CSF sTREM2 variants, which are produced by different TREM2 transcripts. Furthermore, in light of the different amino acid sequences at the C-terminus, additional studies are needed to determine the functions of the two alternatively spliced TREM2 transcripts.

## Functions of TREM2 ectodomain and its soluble form in AD pathology

The shedding of the TREM2 ectodomain, which is triggered by different ligands, is a fundamental process during TREM2-induced cell signaling [32]. The proteolytic shedding of TREM2 results in the release of a soluble protein that regulates the extracellular microenvironment, and the remnant peptides within the transmembrane segment undergo further proteolytic cleavage (by γ-secretase) to induce intracellular signal transduction. Both processes mediate microglial activation in the CNS [51]. Activated microglia may release multiple pro- and anti-inflammatory cytokines to influence the innate immune system, which may affect surrounding cells [52, 53]. TREM2 exerts neuroprotective effects on neurodegenerative diseases not only by producing anti-inflammatory cytokines, but also by promoting the clearance of abnormal proteins and phagocytosis of apoptotic neurons [54]. Since sTREM2 plays a pivotal role in AD pathology and serves as a biomarker in AD [39, 55], we will focus on the TREM2 ectodomain and its cleavage product, sTREM2, for exploring the underlying mechanisms of TREM2 in AD.

### Binding to Aβ and facilitating amyloid plaque phagocytosis

TREM2 has been reported to mediate the phagocytosis of Aβ in the brain [56]. The first step of this process is the association of the TREM2 ectodomain with Aβ fibrils. The V-type immunoglobulin-like region located in the TREM2 ectodomain is the responsible domain for interactions with its ligands. Recently, Zhong and colleagues identified amino acid residues 31–91 of TREM2 as the sequence responsible for its interaction with Aβ by constructing a series of C-terminally truncated recombinant TREM2 proteins and performing Aβ_1–42_ binding assays [57]. Furthermore, the TREM2 ectodomain can also be modified by glycosylation and sulfidation, which increases its affinity for ligands [19].

Aβ, as the most well-studied TREM2 ligand, is a key pathological protein in AD [58]. In vitro studies indicate that variable amyloid filaments exhibit different affinities for the TREM2 ectodomain. In all types of Aβ aggregation, including Aβ_42_ monomers, Aβ_42_ fibrils, and Aβ_40_ monomers, Aβ oligomers have been reported to have the highest affinity for the TREM2 ectodomain [59]. The binding of sTREM2 and Aβ oligomers is characterized by very slow dissociation, which can block the interaction of Aβ oligomers with other ligands. Upon assembly of the TREM2–Aβ complex, microglia are activated to initiate programmed cell phagocytosis in order to clear aggregated proteins [60]. At the same time, TREM2 and sTREM2 settle around Aβ plaques, thereby preventing the plaques from spreading into the surrounding healthy tissues [28, 61–63]. Moreover, microglia around amyloid plaques can be activated to release inflammation-related factors and to initiate anti-plaque cellular signaling. In a recent study, it was demonstrated that the addition of recombinant sTREM2 could decrease Aβ deposition and rescue the behavioral deficits in 5XFAD mice [64]. Taken together, these results indicate that sTREM2 exerts neuroprotective effects by directly affecting the Aβ-induced pathology.

It is worth mentioning that lipoprotein particles, including low-density lipoprotein (LDL), and apolipoproteins, including CLU/APOJ and APOE, are well-studied ligands of TREM2 [65–67]. The results of cell reporter assays indicated that TREM2 receptor-mediated internalization of Aβ was most efficient in microglia when extracellular Aβ was assembled with APOE, LDL, or CLU [66]. APOE, in which the ε4 allele is a strong risk factor for developing late-onset AD [8], has been identified as a novel agonist, as the TREM2–APOE complex can directly modulate AD pathogenesis [68, 69]. Atagi and colleagues showed that APOE promoted the phagocytosis of apoptotic neurons and other abnormal protein aggregates via the TREM2 pathway [70]. Taking into account the findings of Jendresen et al. and the results of a previous study, it seems that amino acids 130–149 of human APOE contain the binding site for the TREM2 ectodomain [66, 68]. Meanwhile, Aβ binding to the lipid-binding region (244–272 aa) of APOE activates TREM2 by the Aβ–APOE complexes found around the amyloid plaques. Furthermore, several studies indicate that APOE variants can also accelerate the course of AD in a TREM2-dependent manner [71, 72]. These results indicate that two AD-related risk genes, TREM2 and APOE, are positively associated, as they both regulate the uptake of Aβ in AD.

### Microglial activation and innate immune system

As a chronic neurodegenerative disease, AD is not just a disease of abnormal protein aggregation, but also one of non-specific neuroinflammation [73]. The innate immune system is aberrantly activated, even before the deposition of Aβ [74]. However, the results on the relationship between protein deposition and neuroinflammation are somewhat conflicting [75, 76]. It has been reported that misfolded Aβ fibrils, which are essentially formed by abnormal innate immune system functioning, can disturb the homeostasis in the brain, whereas other studies contend that protein overload induces an inflammatory response. Presently, it is unclear whether protein deposition or abnormal homeostasis is the initial trigger of AD, although it is clear that the innate immune system is implicated in AD [77, 78].

TREM2 is one of the most crucial factors in regulating the innate immune system during AD progression [45, 79]. There is genetic evidence of the role of TREM2 in the regulation of neuroinflammatory processes in AD [80]. Upon ligand binding, the TREM2 ectodomain undergoes proteolytic cleavage [12], which allows the intracellular fragment of TREM2 to interact with DAP12, thereby activating Syk–PI3K/MAPK signaling and releasing IP3-gated Ca^2+^ stores for phagocytosis [81, 82]. Thus, the shedding of sTREM2 is essential for microglial activation [83]. However, different types of microglia can induce different immune responses [84]. For example, M1 and M2 phenotypes have been reported to participate in neuroinflammation differently. The M1 type is pro-inflammatory and cytotoxic, whereas the M2 type is anti-inflammatory and neuroprotective [85]. Previous studies have indicated that TREM2 overexpression in P301S tau transgenic mice activates M2 microglia to decrease the release of pro-inflammatory cytokines [86, 87]. Therefore, factors that hamper sTREM2 shedding may also inhibit microglial activation and disturb the innate immune system. In addition, the shedding sTREM2 could in turn alter microglial morphology and sustain microglial activation by delivering recombinant Fc-TREM2 to the hippocampi both in wild-type and *Trem2* KO mice [88]. sTREM2 also induces the production of inflammatory cytokines through activating the NF-κB pathway and promotes microglial survival via modulating the activity of Akt/GSK3β/β-catenin signaling pathway in primary microglia from both WT and Trem2-KO mice models [64, 88]. Moreover, sTREM2 has been proved to enhance the clearance of amyloid plaques in an activated microglia-dependent manner, which was also confirmed by 5xFAD mouse model [88]. These results support the notion that sTREM2 mediates microglial-associated inflammatory responses and may act as a protective factor in AD.

However, there are still conflicting opinions on the effects of TREM2 in regulating immune responses, as several studies found that TREM2 can stimulate microglia to release pro-inflammatory cytokines to damage neurons such as IL-1β, TNFα, and IL-6 [45]. In order to explain these inconsistent results, we must understand the course of AD progression and the effect of TREM2 on resident microglia [46]. At the early and middle stages of AD, TREM2 exhibits a protective effect, whereas it shifts toward a detrimental effect by activating the adaptive immune system at the late stages of AD [39, 89]. This viewpoint is consistent with the activation of microglia at different stages of the disease [85]. Meanwhile, sTREM2 is considered to be a decoy receptor because it can trigger inflammatory cytokine production to induce microglial transformation and immune responses to protect against harmful substances during the initial stage of AD [83]. Taken together, these findings demonstrate that TREM2 acts as a protective factor at the early stages of AD by enhancing the protective function of microglia in the brain, but the role of TREM2 at the late stages of AD remains unclear. These findings also suggest that sTREM2 can be a promising therapeutic target by modulating inflammatory responses for AD.

### TREM2 regulates synaptic pruning

Synaptic pruning is the process by which the brain eliminates extra synapses. The synaptic pruning process plays a pivotal role in normal cognition. Multiple in vitro studies found that TREM2 deficiency induces synaptic impairment and axonal dystrophy [90–92]. This is in line with the reduction in the number of microglia in the hippocampus, which significantly associates with synaptic pruning. TREM2 is also involved in synapse regulation through microglia-dependent phagocytosis. Moreover, TREM2 combined with complement proteins, in particular C1q and C3 [93], promoting synaptic elimination of inappropriate connections during mature neural circuit refinement [94]. It is hypothesized that the TREM2 ectodomain or its shedding product, sTREM2, can be an extracellular signal binding the surface receptor of synapses and driving microglia to participate in pruning surplus synapses in order to shape proper brain connectivity [90]. By regulating synaptic pruning, sTREM2 may be involved in signal transmission between neurons. Although a deficit of TREM2 may impair synaptic structure [95] and lead to cognitive impairments in an AD mice model [96], it is still unclear how this process occurs in humans.

### Complex relationship with tau pathology

Presently, the precise regulatory mechanism of TREM2 in tau pathology remains largely unknown. One study found that TREM2 can activate microglia to regulate the inflammatory response by inhibiting pro-inflammatory cytokines, such as TNF-α, IL-1β, and IL-6, in order to decrease the activity of GSK3β and CDK5, and thus, to reduce tau phosphorylation in P301S tau transgenic mice [86]. However, others contend that its loss-of-function can relieve neuroinflammation and protect against neurodegeneration in PS301 transgenic mice [97, 98]. Interestingly, a recent study has demonstrated that TREM2 deficiency exacerbates tau pathology in a tau TREM2 haploinsufficiency mouse model, whereas TREM2 deletion protected against atrophy and reduced inflammatory responses in P301S mice [99]. In general, however, both CSF samples containing sTREM2 and brain tissues from post-mortem AD patients confirm that TREM2 is paralleled by changes in tau/p-tau levels, and even this positive correlation is not influenced by the presence of Aβ [100–102]. These results strongly suggest that TREM2 and sTREM2 play crucial roles in tauopathy.

### Regulating apoptotic neuron clearance

Apart from facilitating the phagocytosis of abnormal proteins, TREM2 also promotes the engulfment of dying cells without eliciting inflammation in CNS [54]. This process is initiated by interactions between the TREM2 ectodomain and apoptotic-associated ligands, including aminophospholipid, phosphatidylserine, and phosphatidylethanolamine [103, 104]. In senescent or apoptotic cells, these ligands are exposed [105], and they can directly bind to the TREM2 ectodomain to induce proteolytic cleavage and phagocytosis of abnormal neurons. This is supported by findings in which TREM2-deficient microglia failed to migrate toward dying neurons in *Trem2*^−/−^ mice [106]. Therefore, the elimination of apoptotic neurons in the absence of inflammation mediated by TREM2 ectodomain-ligand binding is an important process in delaying the progression of AD.

In short, TREM2 can bind various ligands through its extracellular domain to mediate multiple functions. These ligand–receptor interactions can not only help to isolate noxious contents in the brain, but also activate DAP12–Syk signaling, which may induce an inflammatory response. Most studies suggest that TREM2 is a protective factor that can enhance the phagocytic ability of microglia and maintain the homeostasis in the CNS [95]. Moreover, sTREM2 can directly bind pathogenic molecules (e.g., Aβ) or indirectly regulate inflammation [28, 83]. These findings all demonstrate that the extracellular domain of TREM2 and sTREM2 play crucial roles in AD development and may be promising therapeutic targets.

## Variants in TREM2 gene and AD

AD-associated risk variations are identified in the TREM2 ectodomain (Table 1), which is encoded by exon 2 [107]. Most TREM2 mutations affect sTREM2 production and function, which thereby impact the progression of AD [108–110]. The R47H mutation is the most common TREM2 mutation in late-onset AD [111]. The R47H mutation has been reported to triple AD risk in genome-wide association studies, with a 23% faster rate of dementia compared with non-variant carriers [112]. At the cellular level, the R47H mutation disrupts the ligand-binding domain of TREM2, thereby affecting ligand–receptor interactions [108]. A comparison of high-resolution R47H and wild-type TREM2 structures revealed that Arg47 was a key residue in the function of the complementarity-determining region 2 loop, which mediates TREM2 binding with lipids and other ligands [50, 113]. Impaired binding with Aβ fibrils leads to increased accumulation of Aβ plaques and weaker immune activity. Moreover, immunofluorescence studies on brain sections from R47H carriers found fewer activated microglia, leading to a disruption of microglial barrier function [114], consistent with results from the 5XFAD model mice [28]. And R47H-sTREM2 expression could not induce inflammatory responses and enhance microglial survival in primary microglia from WT or TREM2-KO mice [88]. Taken collectively, these findings indicate that the R47H mutation increases the incidence of AD.

**Table 1:** Summary of observed effects of TREM2 variants

Variant	SNP number	TREM2 function	Related disease	CSF sTREM2 level	Shedding of TREM2 ectodomain	Ligand-TREM2 affinity
Q33X	rs104894002	Complete loss	NHD, FTD	Decrease	Decrease	Decrease
Y38C	NA	Complete loss	NHD, FTD	Decrease	Decrease	Decrease
R47H	rs75932628	Partial loss	AD	Increase	Increase	Decrease
R62H	rs374851046	Partial loss	AD	Non	Non	Decrease
T66M	rs201258663	Partial loss	NHD, FTD	Decrease	Decrease	Decrease
D87N	rs142232675	Partial loss	AD	Decrease	Decrease	Decrease
T96K	rs2234253	Partial loss	AD	Decrease	Decrease	Increase
R98W	rs147564421	Partial loss	AD	NA	Decrease	NA
R136Q	rs149622783	NA	AD	Decrease	Decrease	Non
G145T	NA	Partial loss	AD	NA	Decrease	Decrease
E151K	rs79011726	Partial loss	AD	NA	NA	NA
H157Y	rs2234255	NA	AD	NA	Increase	Non
W191X	rs2234258	Partial loss	AD	Decrease	NA	NA
L211P	rs2234256	Partial loss	AD	Decrease	NA	NA

*FTD* frontotemporal dementia, *NHD* Nasu-Hakola disease, *NA* not applicable

The R62H mutation is another well-studied point that increases the risk for late-onset AD [17, 115]. It also reduces microglial activation and inhibits immune responses, although it increased TREM2 expression in the brain of AD patients [115]. Some other AD-associated variants of TREM2 are p.D87N, p.T96K, and p.H157Y [19, 26, 116, 117], and these variants were found by examining the genetic variability a large AD populations. Other TREM2 ectodomain variants (e.g., T66M, Y38C, and Q33X) were found to be more associated with NHD than AD (Table 1) [118]. These variants change the systemic expression level of TREM2 and influenced the production of sTREM2 in the whole body, which not only deteriorated microglial function in the brain, but also caused bone impairment. For example, the T66M mutation can decrease the sTREM2 level in the brain but increase the sTREM2 level in the plasma [42, 118]. Furthermore, dementia in T66M variant carriers is more likely an accompanied symptom rather than cardinal symptom [119]. The H157Y variant is also thought to increase the production of sTREM2 by modifying cleaved sites [27]. Thus, further studies are needed to identify the functional changes induced by TREM2 ectodomain mutants.

In addition, the sTREM2 level in the CSF is also susceptible to TREM2 gene variants [42, 117, 118]. In 2016, Piccio et al. analyzed the impact of the AD-associated variants on sTREM2 in the CSF [42] and showed that both AD-associated variants (R62H, L211P, and W191X) and NHD-associated variants (R136Q, D87N, Q33X, and T66M) can decrease the concentration of sTREM2 in the CSF compared to non-carriers, suggesting that these mutations reduce membrane protein turnover. Interestingly, the R47H mutation, which increased sTREM2 expression in the CSF, had no impact on sTREM2 expression in the brain of AD patients [50]. It is possible that the R47H mutation affects ligand binding, thereby altering the proteolysis of TREM2 [50, 110]. In light of these contradictory findings in the CSF and brain, further studies are needed to understand the metabolism of sTREM2 and its circulation in the CSF.

## (s)TREM2 as therapeutic targets

At present, there is still no effective disease-modifying therapeutics for AD. As aforementioned, the extensive genetic and biomarker evidences suggest a central role of TREM2 in AD pathogenesis [111, 115]. Thus, several groups attempted to design therapeutic strategies targeting TREM2 or sTREM2 for AD, including increasing TREM2 or sTREM2 level, activating TREM2, etc. Recently, Lee et al. conducted a gain-of-function genetic approach to generate TREM2 transgenic mice expressing human TREM2 in microglia [120], in order to determine whether increased TREM2 expression had impact on the development of AD. The results indicated that elevated TREM2 gene dosage reprogramed the transcriptional responses and modified the morphology of microglia. Overexpression of TREM2 also reduced the density of plaques and improved behavioral outcomes in AD mice. On the other hand, heat shock protein 60 (HSP60), a mitochondrial chaperone, has been identified as a specific TREM2 agonist [121]. Hsp60 activated TREM2-associated signaling pathway and enhanced microglial phagocytosis via binding to the immunoglobulin region of TREM2 ectodomain. Although the underlying biochemical pathway mediated by Hsp60 is still unclear, it has been widely accepted as a potential target for other diseases including atherosclerosis and cancer [122–124]. Hence, the pharmaceutical experiences of Hsp60 applied in other diseases should be referred to explore the potential strategies to treat AD.

Due to the crucial roles of sTREM2 in modulating microglial function, the direct injection of purified sTREM2 into the hippocampus in AD mouse models has been performed to estimate its therapeutic effect on AD [64]. Recombinant sTREM2 reduced amyloid plaque load in a mouse model of AD. In addition, TREM2-activating antibodies reversed the negative pleiotropic effects of R47H mutant TREM2 on microglia [125]. These results suggest that TREM2 ectodomain is required for microglial functions in vivo. On the contrary, another group demonstrated that an antibody against the stalk region of TREM2 inhibited its shedding from cell surface [126]. This antibody showed protective effect against AD pathology by modulating TREM2 function and promoting microglia transitioning to the disease-associated state [126]. Thus, further investigations are needed to illustrate the effect of sTREM2 shedding on AD.

## Conclusions and perspectives

TREM2 ectodomain, as a receptor expressed on the surface of microglia, undergoes proteolysis to generate sTREM2 in the brain. Most pathophysiological effects of TREM2 begin with the production of sTREM2 following changes of local microenvironment in CNS [51]. sTREM2 is also a reliable biomarker of AD [39]. On the one hand, sTREM2 exerts a neuroprotective effect against AD by stimulating the innate immune system. It drives microglia to migrate toward Aβ fibrils and abnormal neurons to facilitate phagocytosis [10, 127]. On the other hand, sTREM2 plays a critical role in tau phosphorylation. Thus, sTREM2 might contribute to the transport, delivery, or metabolism of two important AD-associated proteins, Aβ and tau.

Despite the in vitro and in vivo evidence, the role of sTREM2 in the development of AD is still in dispute. Most researchers hold sTREM2 exerts a neuroprotective effect by improving the clearance effect of microglia [128], whereas sTREM2 can also trigger microglia to release pro-inflammatory cytokines, which may adversely affect neuronal function [129]. Studies have also reported that different body fluids, such as the CSF and blood, contain different levels of sTREM2 at different stages of AD progression [11]. At this point, further studies are needed to understand the precise functions of sTREM2 and the role of this protein in the pathology of AD. This information is needed in order to arrive at a novel therapeutic strategy, which should contribute to our understanding of AD.

## Data Availability

Not applicable

## Abbreviations

AD: Alzheimer’s disease
TREM2: Triggering receptor expressed on myeloid cells
sTREM2: Soluble TREM2
CSF: Cerebrospinal fluid
CNS: Central nervous system
Aβ: β-amyloid peptide
APOE: Apolipoprotein
APP: Amyloid precursor protein
PSEN1: Presenilin 1
PCR: Polymerase chain reaction
ADAMs: Disintegrin and metalloproteases
MS: Multiple sclerosis
FTD: Frontal temporal dementia
LBD: Lewy body dementia
moDCs: Monocyte-derived dendritic cells
SYK: Spleen tyrosine kinase
PI3K: Phosphoinositide 3-kinase
MAPK: Mitogen-activated protein kinase
IP3: Inositol trisphosphate
Akt: Protein kinase B
DAP12: DNAX-activating protein of 12 kDa
GSK3β: Glycogen synthase kinase β
CDK5: Cyclin-dependent kinase 5
TNF-α: Tumor necrosis factor-α
IL-1β: Interleukin-1β
IL-6: Interleukin-6
C1q: Complement component 1q
C3: Complement component 3
